# Menstrual cycles during COVID-19 lockdowns: A systematic review and meta-analysis

**DOI:** 10.3389/frph.2022.949365

**Published:** 2022-08-09

**Authors:** Melissa Chao, Carlo Menon, Mohamed Elgendi

**Affiliations:** ^1^Faculty of Medicine, University of British Columbia, Vancouver, BC, Canada; ^2^Biomedical and Mobile Health Technology Laboratory, Department of Health Sciences and Technology, Zurich, Switzerland

**Keywords:** COVID-19, menstruation, menstrual disturbance, menstrual change, pandemic, lockdowns

## Abstract

Coronavirus disease 2019 lockdowns produced psychological and lifestyle consequences for women of reproductive age and changes in their menstrual cycles. To our knowledge, this is the first systematic review to characterize changes in menstrual cycle length associated with lockdowns compared to non-lockdown periods. A search on 5 May 2022 retrieved articles published between 1 December 2019, and 1 May 2022, from Medline, Embase, and Web of Science. The included articles were peer-reviewed observational studies with full texts in English, that reported menstrual cycle lengths during lockdowns and non-lockdowns. Cross-sectional and cohort studies were appraised using the Appraisal tool for Cross-Sectional Studies and the Cochrane Risk of Bias Tool for Cohort Studies, respectively. Review Manager was used to generate a forest plot with odds ratios (OR) at the 95% confidence interval (CI), finding a significant association between lockdown and menstrual cycle length changes for 21,729 women of reproductive age (OR = 9.14, CI: 3.16–26.50) with a significant overall effect of the mean (*Z* = 4.08, *p* < 0.0001). High heterogeneity with significant dispersion of values was observed (*I*^2^ = 99%, τ = 1.40, χ^2^ = 583.78, *p* < 0.0001). This review was limited by the availability of published articles that favored high-income countries. The results have implications for adequately preparing women and assisting them with menstrual concerns during lockdown periods.

## Introduction

The global spread of coronavirus disease 2019 (COVID-19), caused by severe acute respiratory syndrome coronavirus 2 (SARS-CoV-2), has become a severe public health crisis. The global pandemic has been associated with high rates of infection and mortality worldwide (1). To contain the spread of COVID-19 in the population, a variety of preventative policies were enacted (2).

Some common preventive policies have included quarantine, which restricts the movement (typically for 2 weeks) of exposed people or those who have traveled to an affected area, reducing physical interaction with others in the community, including avoiding social gatherings, wearing face masks, staying six feet (two meters) from other people, avoiding common private or public spaces, and working from home when possible, and isolation (i.e., infected people isolating to protect non-infected people). Meanwhile, lockdown—the most extreme public health measure—restricts the movement of people when a fraction of the population has become infected. This can include shutting down of schools, universities, public transport, domestic and international travel, places of worship, and places of social gathering, while hospitals remain open (2).

It is well documented that COVID-19 pandemic lockdowns are associated with many respiratory health concerns, but there is a growing understanding that the health consequences of pandemic lockdown policies have multiple effects on the population (3–6).

The COVID-19 pandemic has invariably produced many negative mental health consequences, including increased stress levels due to only the fear of contracting or spreading COVID-19 to others, but also disruptions to work or school, growing levels of unemployment, and other financial constraints (7–9). Other consequences, include depression, anxiety, and insomnia (7). However, COVID-19 lockdowns have unequal mental health effects on the population (7). Specifically, women of reproductive age are reported to have increased stress levels due to lockdowns (10, 11).

The COVID-19 pandemic has exacerbated many menstrual challenges for women of reproductive age despite growing attention to women's reproductive and sexual health. It is therefore becoming increasingly important to examine the variability of menstrual cycles in the context of COVID-19. High levels of perceived stress are associated with irregular cycles, so it is conceivable that the pandemic has had profound impacts on menstrual cycle regularity (12).

The typical menstrual cycle length is 28 days +/- 7 days, with a typical menstrual duration of between 2–7 days (13). However, menstrual cycles may be disrupted by high levels of stress, resulting in irregularities (14). Irregular menstrual cycles include changes in frequency, volume, regularity, duration, severity of menstrual-related symptoms, and spotting/intermenstrual bleeds (14, 15).

The endometrium is a multicellular tissue in the uterus that is the target of sex steroid regulation. The hypothalamic-pituitary-gonadal axis (HPG axis) maintains control of circulating reproductive hormones, which in turn regulate reproductive organs. The hypothalamus secretes gonadotropin-releasing hormone (GnRH), which causes the anterior pituitary to release gonadotropins follicular-stimulating hormones (FSH) and luteinizing hormone (LH). Subsequently, FSH and LH stimulate the growth of the ovarian follicle with secretion of estrogen during the follicular phase, and following ovulation, they stimulate the release of progesterone during the luteal phase (16). In a progesterone-primed endometrium, the lack of fertilization results in progesterone withdrawal. This triggers menstruation—the shedding of endometrium tissue (17).

Many historical global stressors have been associated with changes in the menstrual cycle for women of reproductive age (18, 19). One particular concern is functional hypothalamic amenorrhea (FHA), a disorder characterized by chronic anovulation caused by a disruption of the HPG axis (20). One subclassification of FHA, stress-related FHA, is caused by high stress levels that alter the HPA axis (21). This increases corticotrophin-releasing hormone and cortisol levels, which decrease GnRH levels and have downstream overall reduction in estrogen levels (21). The state of hypoestrogenism may have implications for homeostasis of the entire body, including the impairment of gonadal function and the absence of menstruation (22, 23).

Another conceivable link exists with abnormal uterine bleeding, menstrual flow that deviates from the normal volume, duration, regularity, or frequency, which is inclusive of heavy menstrual bleeding and intermenstrual bleeding (24). Similar to FHA, stress-related disruptions are related to poor hemostatic and vasoconstrictive capabilities of endometrium that result in abnormal uterine bleeding (24, 25). Mood and anxiety disorders, possibly due to hormone level fluctuations, as well as stressful events and psychiatric disorders can trigger irregular menstruation (25, 26).

Another menstrual disorder is dysmenorrhea, defined as a severe, painful, cramping sensation in the lower abdomen accompanied by other premenopausal symptoms, such as sweating, headaches, nausea, vomiting, diarrhea, and more (27). Studies have demonstrated that depression and stress increase the risk of dysmenorrhea, and there is a positive association between stress and dysmenorrhea and depression and menstrual pain (27). Taken together, COVID-19 pandemic lockdowns have invariably worsened psychological health, including increasing stress and other mental health issues; therefore, it is conceivable that these major changes have resulted in various menstrual health concerns for women of reproductive age (27).

Recent studies have reported that COVID-19 lockdown measures have a wide breadth of sexual and reproductive health consequences in women of reproductive age (28–30). One recent study reported that women experienced more frequent irregular cycles during the pandemic (31, 32). However, a bidirectional relationship does exist: menstrual symptoms also have strong negative impacts on daily activities and well-being, inducing problems such as poor mental health, reduced productivity at work, and stress on health systems (8, 9, 33–35).

As the COVID-19 pandemic continues to present an overwhelming global public health crisis, many women of reproductive age are experiencing pandemic lockdowns. As such, these lockdowns present menstrual health consequences for this population, making it critical to study the consequences of pandemic lockdowns on menstrual cycles. To our knowledge, no systematic review has reviewed the impact of the COVID-19 pandemic on the menstrual cycle patterns of women of reproductive age, making this the first study to do so and recommend future avenues of investigation.

## Methods

The review process for this systematic review comprised five phases: (1) potential articles were identified through database and manual searches, (2) articles were reviewed for eligibility according to inclusion and exclusion criteria, (3) eligible articles underwent quality appraisal according to the Appraisal tool for Cross-Sectional Studies (AXIS) or Cochrane Tool to Assess Risk of Bias in Cohort Studies, (4) data on outcomes of interest from eligible original articles were extracted, and (5) data analysis was performed. The search protocol was not registered.

### Database and manual searches for articles

A protocol developed in consultation with a librarian from the University of British Columbia identified the search terms used in this study. The search for COVID-19 lockdowns included the following terms: “COVID-19,” “SARS-CoV-2,” “nCoV-2019,” “coronavirus infections,” “viral pneumonia,” and “pandemics.” The menstruation search terms included “menstruation,” “menstruation disturbance,” “menses,” “menstrual flow,” “menstrual discharge,” “menorrhea,” “menarche,” and “monthlies” (Supplementary Information). Search terms were deployed on 5 May 2022 to identify peer-reviewed articles published between 1 December 2019, and 1 May 2022, from Medline (OVID), Embase (OVID), and Web of Science.

Following the database search, reference lists of relevant eligible articles were manually searched to identify additional eligible articles. The review and analysis of the articles reported were conducted according to the Preferred Reporting Items for Systematic Reviews and Meta-Analyses guidelines (36, 37).

### Inclusion and exclusion criteria

The inclusion criteria were journal articles published between 1 December 2019, and 1 May 2022, found in Medline, Embase, and Web of Science that had full text available, were available in English, were published in a peer-reviewed journal, discussed premenopausal, menstruating women, and specifically reported on the length of the menstrual cycle during both the COVID-19 pandemic lockdown and non-lockdown periods. The article type was restricted to original articles that described observational studies. However, articles were not excluded based on geographical location, patient age, COVID-19 status, pregnancy status, ovulatory status, reproductive history, or any other patient factors.

The exclusion criteria were articles that did not have abstracts, lacked a full text available in English, were not published in a peer-reviewed journal, included opinion pieces, letters, commentaries, guidelines, and simulations/modeling, were published outside the timeframe 1 December 2019, and 1 May 2022, and did not discuss potential menstrual cycle changes before and during lockdowns.

### Article review

Following the article search, all identified articles were collected and uploaded into the Covidence tool for systematic reviews, and duplicates were automatically removed (38). Subsequently, the review phase comprised title and abstract screening, and full-text review. The review was completed in a blinded independent manner by MC and ME to avoid selection bias, and disagreements were discussed until a consensus was reached. During the title and abstract screening and full-text review, the articles were filtered according to the inclusion and exclusion criteria.

### Critical appraisal of cross-sectional studies

To critically appraise the cross-sectional studies included, the AXIS tool, which was specifically designed for such tasks, was deployed (39). The AXIS tool helped ensure high quality and low bias in the study design of each cross-sectional study included (39).

This 20-item appraisal tool was applied to each included study to assess the clarity and appropriateness of the aims/objectives, justification for the sample size and populations, representativeness of the populations, measures to address non-responders, appropriateness of risk factors, outcome variables, statistical tests, sufficiency of the methodological description, adequacy of the reported data, sufficiency of addressing concerns with non-responders, internal consistency of results, sufficiency of the results, justification of the discussion and conclusions, limitations of the study's findings, and ethical considerations (39).

For each of the 20 items, each cross-sectional study was scored, and a binary response was given. For every item, a score of one was given for items with a low risk of bias, and zero was given for a response with a high risk of bias. The articles were then categorized into quartiles by how many items of the AXIS criteria were met: Q1 = 15–20 items; Q2 = 10–14 items; Q3 = 5–9 items; Q4 = 0–4 items (39). Only cross-sectional studies that fell within the first quartile were included in this systematic review. The appraisal tool was completed by MC and ME in a blind and independent manner, and any disagreements were discussed until resolved.

### Critical appraisal of cohort studies

To critically appraise the cohort studies included the Cochrane Tool to Assess Risk of Bias in Cohort Studies was used to ensure high quality and low bias in the study design of each included cross-sectional study (40, 41).

This eight-item appraisal tool was applied to each included study to assess the selection of exposed and non-exposed cohorts, confidence regarding the assessment of exposure, confidence that the outcome of interest was not present at the start, matching of exposed and unexposed cohorts for all variables, confidence in the prognosis factor, confidence in the assessment of the outcome, adequacy of follow-up, and similarity in co-interventions between groups (40).

For each of the eight items, each cohort was scored as either “definitely yes” (low risk of bias) for four points, “probably yes” for three points, “probably no” for two points, or “definitely no” (high risk of bias) for one point (40). For each article, the points were tallied. The articles were categorized into quartiles as follows: Q1 = 24–32 points; Q2 = 16–23 points; Q3 = 8–15 points; and Q4 = 0–7 points. Only cohort studies that fell within the first quartile were included in this systematic review. The appraisal tool was completed by MC and ME in a blind and independent manner, and any disagreements were discussed until resolved.

### Data extraction for outcomes of interest

During the data extraction phase, the reviewers collected 10-item Covidence data, populated each of the 10 items for all included articles, and exported the populated form into Microsoft Excel (38, 42). The data collection captured outcomes of interest, as described in both the article and its corresponding Supplementary Information.

The research question was to investigate how the menstrual cycle length of reproductive age women changes during pandemic lockdowns compared to before pandemic lockdowns. As such, the primary outcome of interest was the change in menstrual cycle length during pandemic lockdowns compared to non-lockdown periods. In this context, changes to menstrual cycle lengths are defined as cycles that are longer or shorter than usual for the patient, whereas menstrual irregularity is broader, encompassing changes in the volume and duration of menstruation, amenorrhea, and changes to menstrual or premenstrual symptoms (10, 43, 44). Data were collected on the number of women whose cycle lengths had changed during the lockdowns, remained unchanged during the lockdowns, changed before the lockdowns, and remained unchanged before the lockdowns.

To handle the unpopulated data fields, the number of women was calculated based on the given values. If, for example, the number of women with cycle changes during lockdown was missing, while the total number of women and the number of women without cycle changes during lockdown were presented, then the missing value was calculated using subtraction.

### Statistical analysis

Following the article review, the data on the outcomes of interest were analyzed using Review Manager (RevMan) 5.4.1 software (45). A forest plot was used to visually depict the odds ratios (OR) for each included study. A funnel plot was developed using RevMan to determine the sensitivity of the review, and statistical tests in RevMan were deployed for meta-analysis.

The ORs and 95% confidence interval (CI) were calculated for each study to determine the odds that the primary outcome was associated with the exposure cohort (46). A random effects model for discrete data was employed. The *I*^2^ test, τ^2^ test, and χ^2^ test were deployed to quantify the level of heterogeneity between studies in the meta-analysis, and a *p*-value was calculated to level the significance of the heterogeneity (47).

Weighted mean differences were used to analyze statistical data effectiveness, and a 95% CI was calculated. Once the population mean was determined, the *Z*-test statistic was employed to test the reduction of uncertainty in past events, and the *p*-value was calculated (48).

## Results

### Search results

**Figure 1:**
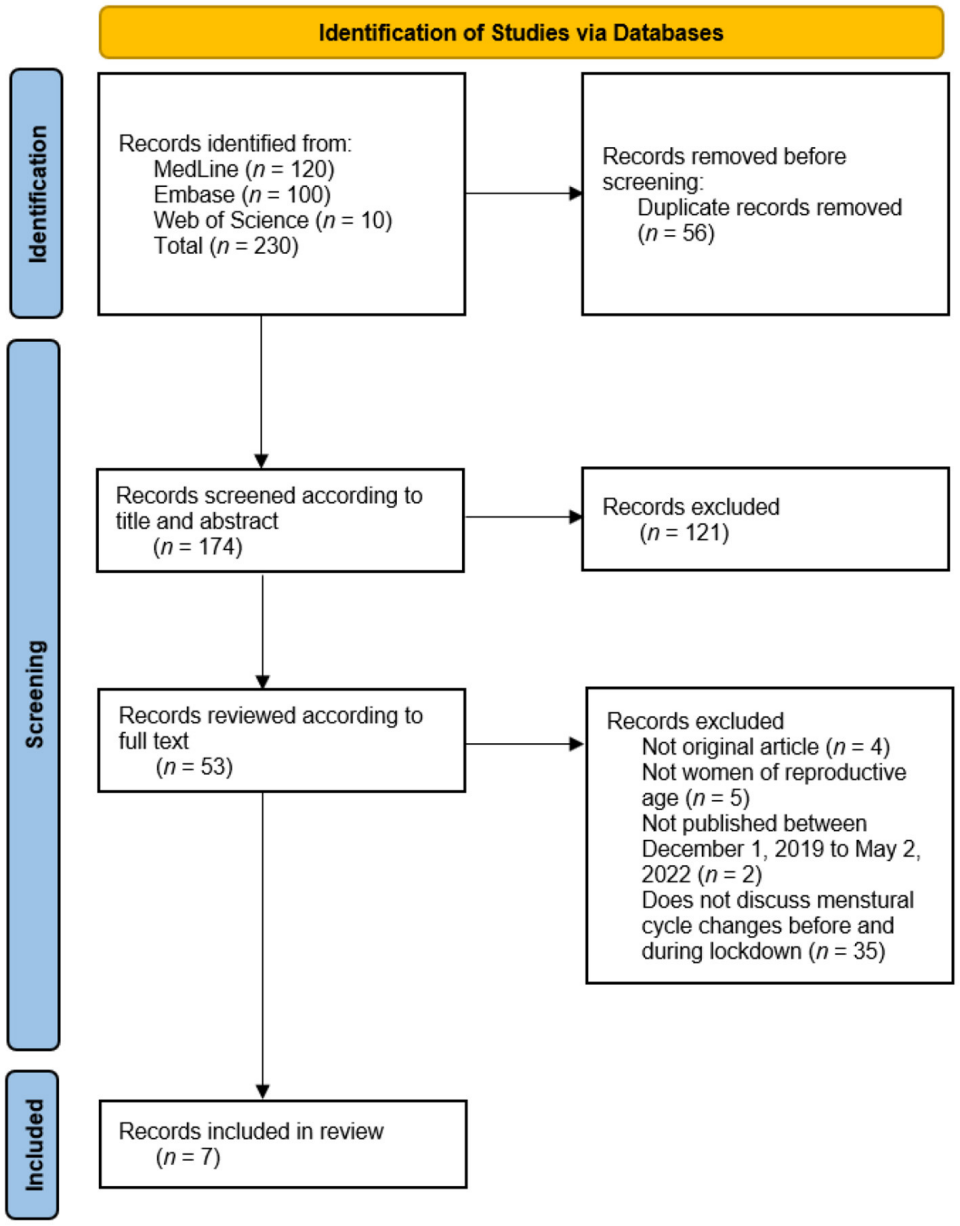
PRISMA flow diagram for systematic reviews.

In total, 230 articles were identified through a search of three databases (120 articles from Medline, 100 articles from Embase, and 10 articles from Web of Science). After 56 duplicate articles were removed, the abstracts and titles of the remaining 174 articles were screened, producing 53 articles for full-text review. All included papers were hand-searched for references, resulting in no additional articles identified for title and abstract screening. Finally, after a full-text review, seven articles were included in this review, and they described 21,729 women: 21,729 from extant lockdown and 21,728 women from non-lockdown (Figure 1).

### Quality assessment results

All six articles included in this systematic review fell in the top quartile during the screening process, where cohort studies were screened using the Cochrane Tool for Cohort Studies, and cross-sectional studies were screened using the AXIS tool (39, 40). They comprised different observational study designs: four cross-sectional studies and two cohort studies. Of the six articles, the majority were published in high-income countries, such as Ireland, United States, and Great Britain. Two were published in low- and middle-income countries (LMIC, two from Turkey). Five of the six studies were published in 2021, while one was published in 2022. The AXIS quality assessment scores for all included articles are presented in Table 1. The full quality assessment is presented in the Supplementary Information.

**Table 1:** Characteristics of all included studies.

**ID**	**Publication year**	**Authors**	**Title**	**Study design**	**Country**	**Age (mean years, range)**	**Sample size**	**AXIS quality appraisal score**	**Cochrane tool for cohort studies**
1	2021	Buran and Gercek (44)	Impact of the awareness and fear of COVID-19 on menstrual symptoms in women: A cross-sectional study	Cross-sectional	Turkey	27.1, 18–42	125	18/20	N/A
2	2021	Nguyen et al. (32)	Detecting variations in ovulation and menstruation during the COVID-19 pandemic, using real-world mobile app data	Cohort	Great Britain, United States, Sweden, other countries	32.5, N/A	18,076	N/A	27/32
3	2021	Ozimek et al. (43)	Impact of stress on menstrual cyclicity during the coronavirus disease 2019 pandemic: A survey study	Cohort	United States	32.5, 18–45	210	N/A	28/32
4	2021	Phelan et al. (11)	The impact of the COVID-19 pandemic on women's reproductive health	Cross-sectional	Ireland	36.7, 15–54	1,031	18/20	N/A
5	2021	Takmaz et al. (49)	The impact of COVID-19-related mental health issues on menstrual cycle characteristics of female healthcare providers	Cross-sectional	Turkey	29.5, 18–40	952	19/20	N/A
6	2022	Maher et al. (10)	Female reproductive health disturbance experienced during the COVID-19 pandemic correlates with mental health disturbance and sleep quality.	Cross-sectional	Ireland	N/A, 29–38	1,335	19/20	N/A

AXIS, Appraisal tool for Cross-Sectional Studies; COVID-19, Coronavirus Disease 2019; N/A, not applicable.

### Cycle length

**Figure 2:**
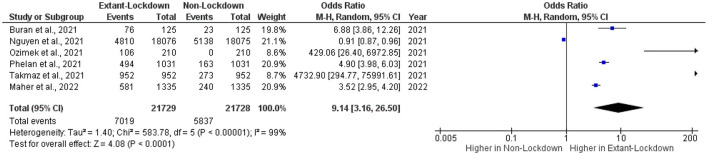
Effect of COVID-19 pandemic lockdowns on cycle length changes.

Overall, 21,729 women of reproductive age were included in the extant lockdown group, and 21,728 women were included in the non-lockdown group (Figure 2). The OR and 95% CI were calculated for each included article, ranging in value from 0.91–4,732.90. One study had ORs under one, while five had ORs greater than one. Interestingly, studies with ORs under one had narrow 95% CIs, while studies with higher ORs had broader 95% CIs.

The pooled OR was 9.14 (CI: 3.16–26.50), indicating an association between lockdown policies and changes in the menstrual cycle. The heterogeneity of values in the included studies was assessed, finding a statistically significant dispersion of values (τ^2^ = 1.40, χ^2^ =583.78, *df* = 5, *I*^2^ = 99%, *p* < 0.0001). The overall effect of the mean was statistically significant (*Z*-test = 4.08, *p* < 0.0001).

## Discussion

### Result heterogeneity

With only six included studies and high heterogeneity, as indicated by the statistical tests, a small sample size bias may exist and may be responsible for the symmetrical distribution. This heterogeneity may be due to the variability in the sample sizes for each included study. The skew toward smaller standard error values indicates high levels of precision, which were likely due to publication bias stemming from articles publishing similar results on menstrual cycle length changes and neglecting to publish articles with no change in cycle length.

Despite this heterogeneity in the included samples, there was a positive association between pandemic lockdowns and menstrual cycle length change for 21,729 women in the extant-lockdown group compared to 21,728 women in the non-lockdown group.

### Analysis and interpretation of included studies

The six included articles, including the main findings and contributions to the literature, are summarized here. Buran and Gercek (2021) investigated the impact of awareness and fear of COVID-19 on menstrual symptoms in one group of women before and during pandemic lockdowns. They found that a higher awareness of COVID-19 was related to changes in menstrual cycle changes but not menstrual symptoms (e.g., pain, heavy menstrual bleeding, and premenstrual syndrome) (44, 50).

Nguyen et al. (32) studied menstrual cycles of women from Great Britain, the United States and other countries using cycle tracking app data. They observed that menstrual irregularities were more prevalent before the pandemic (32).

In 2021, Ozimek et al. (43) studied menstrual cycles during the early months of the COVID-19 pandemic; observing many changes in menstrual cycles, including cycle length, menstruation duration, changes in symptoms, and significant perceived stress. Subsequently, Phelan et al. (11) reported on various menstrual changes experienced by women, finding that nearly half of the subjects experienced changes in their menstrual cycle that were observed since the start of the pandemic. Agreeing with other included articles, these subjects also reported significant lifestyle and mental health changes that impacted their overall reproductive health.

Takmaz et al. (49) distributed an online questionnaire to female healthcare practitioners comparing lifestyle changes and mental health changes between women with regular vs. irregular menstrual cycles. Consistent with Buran and Gercek, Ozimek et al. (43), and Phelan et al. (11), Takmaz et al. (49) also found a correlation between irregular menstrual cycles and mental health concerns (44). Finally in 2022, a survey distributed on social media platforms conducted by Maher et al. (10) measured changes in mental health, lifestyle changes, and various other menstrual health indicators. This research group studied the characteristics of women under normal circumstances compared to under pandemic lockdown. In agreement with other included articles, Maher found a correlation between reproductive health issues and decreased psychological status.

### Implications for quality of life

The increased odds of irregular menstrual cycle length in women experiencing pandemic lockdowns echo the various quality-of-life concerns that existed for women of reproductive age before the pandemic but were exacerbated during the pandemic.

Most importantly, it is well documented that irregular menstrual cycles negatively impact the quality of life of women of reproductive age (51–54). Even before the pandemic, menstrual symptoms interfered with the daily activities of a large fraction of women, and such menstrual irregularities were associated with lower education levels and household incomes, which serve to highlight certain social inequities (33, 55).

However, lockdown policies during the pandemic may have exacerbated social concerns for women of reproductive age with irregular menstrual cycles. A compounding feedback loop may exist between menstrual irregularities and stress, thus decreasing the quality of life of many women. Stressful situations may lead to irregular menstrual cycles, which in turn may have downstream impacts on stress for some women (31, 56). For example, work or family responsibilities during the pandemic may have increased stress, contributing to menstrual irregularities and worsening health outcomes for women (56). Furthermore, negative financial considerations, such as loss of employment or loss of benefits, may have also been stressors and negatively impacted women's sexual health (56).

An additional bidirectional relationship exists between access to healthcare services and negative sexual health outcomes. Interruptions in women's regular sexual and reproductive healthcare may contribute to menstrual cycle disturbances, which may, in turn, pose further barriers to seeking healthcare (57).

However, the flexibility afforded by working from home or lockdown policies may have assisted some women with menstrual irregularities, allowing them the comfort of continuing to work from their own homes. For some women with menstrual issues, work-from-home policies may have increased their access to menstrual products, thus improving their condition.

### Implications for healthcare and public health

Characterizing menstrual irregularities across populations provides insights into menstrual inequities during stressful public health situations and is the first step in addressing these inequities. These findings are dually applicable to the healthcare and public health fields.

In clinical applications, this article may make clinicians more aware of menstrual cycle irregularities and any associated health complications that may arise during the COVID-19 pandemic. Increased awareness of this clinical presentation may allow clinicians to easily identify health concerns for women with irregular menstrual cycles and to plan for the care of women with menstrual concerns.

Additionally, public health leaders may use this information to design policies or programs to support women of reproductive age. For example, public health initiatives that have identified social inequities in their communities may seek to increase access to sexual and reproductive care for women experiencing menstrual concerns during the pandemic.

### Limitations of this review

This systematic review has several limitations. First, it could not account for the ongoing nature of the pandemic, and the resulting policy changes that may impact the lockdown duration degree or any other variation. Data were collected irrespective of geographical location, phase of the pandemic, and lockdown policies, which limits the generalizability of the study to a limited resolution into various phases.

Second, this review did not capture various nuances in menstrual cycle changes, such as the degree of menstrual cycle changes, any associated sexual or reproductive health concerns, or the demographics of women who experience menstrual cycle irregularities.

Third, awareness of pandemic-related menstrual cycle changes is only beginning to emerge, making it a new research avenue. As such, this systematic review was limited by the availability of scarce data on menstrual cycle changes.

Fourth, due to its strict inclusion criteria, this systematic review has limited generalizability to the global population of women of reproductive age. As searches were limited to published articles in English, this created a publication and language bias. Subsequently, this study primarily reflects the experiences of high- or middle-income countries even though the majority of perceived stress is experienced in low-income countries. This also favors the reporting of certain ethnicities over others, which may not be representative of global populations (58).

Therefore, this systematic review captured only select populations, often representing populations from high- or middle-income countries with access to healthcare services or COVID-19 testing services. In addition to limitations in the population, there was a reporting bias in the selection of included articles because observational studies in the literature often neglected to report normal cases (i.e., unchanged menstrual cycles), leading to an overestimation of the effect size.

### Future works

As this systematic review only investigated cycle length, future systematic reviews should subcategorize various changes in menstrual patterns, including irregularities in menstrual and perimenstrual symptoms, menstrual volume, and menstruation duration to better characterize and improve conditions for menstruating women. In addition, future studies should consider how menstrual cycle irregularities may have downstream impacts on women's reproductive and sexual health. As various phases of the pandemic have imposed different lockdown policies, further details regarding changes in the menstrual cycle during different phases may provide further insight.

To overcome issues with data availability in LMIC, where the majority of perceived stress occurs, future studies should explore unpublished work and papers in non-English languages from a wider range of data sources. As various confounding variables exist for the circumstances of women of reproductive age, a lower proportion of women may have affected menstrual cycles; thus, future studies may compare the effects of COVID-19 pandemic lockdown policies on menstrual cycles to the effects of other recent outbreak lockdowns, such as the Ebola outbreak.

This systematic review considered cycle length, irrespective of infection or vaccination status. Future review studies may investigate the impact of infection and vaccination status to lessen the burden on menstruating women of reproductive age. Finally, future reviews may investigate whether the cycle lengths returned to normal after the pandemic.

## Conclusion

This systematic review synthesizes the growing body of evidence on the presence of menstrual disturbances during COVID-19 pandemic lockdowns, specifically finding an association between changes in menstrual cycle length for women of reproductive age. This finding has implications for both public health leaders and clinicians in preparing and adequately treating women of reproductive age with menstrual concerns. Further investigations on the impact of vaccination and SARS-CoV-2 infection status on menstrual cycles are needed to characterize the diverse experiences of women of reproductive age during the COVID-19 pandemic.

## Data availability statement

The original contributions presented in the study are included in the article/Supplementary material, further inquiries can be directed to the corresponding author.

## Author contributions

MC, CM, and ME conceived the study. All authors approved the final manuscript.

## Funding

Open access funding provided by ETH Zurich.

## Conflict of interest

The authors declare that the research was conducted in the absence of any commercial or financial relationships that could be construed as a potential conflict of interest.

## Publisher's note

All claims expressed in this article are solely those of the authors and do not necessarily represent those of their affiliated organizations, or those of the publisher, the editors and the reviewers. Any product that may be evaluated in this article, or claim that may be made by its manufacturer, is not guaranteed or endorsed by the publisher.
