# Supplementary material for: Menstrual cycles during COVID-19 lockdowns: A systematic review and meta-analysis
Source: Front Reprod Health. 2022 Aug 9;4:949365. doi: 10.3389/frph.2022.949365 (PMC9580671; doi:10.3389/frph.2022.949365)
Supplement: Supplementary file 1 [file Data_Sheet_1.pdf]

SUPPLEMENTARY INFORMATION

OVID Search Terms

{(Coronavirus infection/ OR COVID-19/ OR SARS-CoV-2/ OR pneumonia, viral/ OR pandemics/ or coronavirus/) OR [(COVID-19 OR coronavirus* OR SARS-CoV-2 OR nCoV-2019 OR viral pneumonia* OR pandemic* OR coronavirus*).mp. (mp = title, abstract, original title, name of substance word, subject heading word, floating sub-heading word, keyword heading word, organism supplementary concept word, protocol supplementary concept word, rare disease supplementary concept word, unique identifier, synonyms)]} AND {(Exp Menstrual Disturbances/ or exp Menstruation/ or exp Menarche/) OR [(menstrua* or menstrual disturbance* OR menstrual change* OR menstrual flow* OR menstrual discharge* OR menses* OR menorrhea* OR menorrhoea* OR menarche* or monthlies*).mp. (mp=title, abstract, original title, name of substance word, subject heading word, floating sub-heading word, keyword heading word, organism supplementary concept word, protocol supplementary concept word, rare disease supplementary concept word, unique identifier, synonyms)]}

Appraisal Tool for Cross-Sectional Studies (AXIS) Tool

**Article ID:**	**1**	**4**	**5**	**6**
1. Were the aims/objectives of the study clear?	1	1	1	1
2. Was the study design appropriate for the stated aim(s)?	1	1	1	1
3. Was the sample size justified?	1	1	1	1
4. Was the target/reference population clearly defined? (Is it clear who the research was about?)	1	1	1	1
5. Was the sample frame taken from an appropriate population base so that it closely represented the target/reference population under investigation?	1	1	1	1
6. Was the selection process likely to select subjects/participants that were representative of the target/reference population under investigation?	0	1	1	1
7. Were measures undertaken to address and categorise non-responders?	0	0	0	0
8. Were the risk factor and outcome variables measured appropriate to the aims of the study?	1	1	1	1
9. Were the risk factor and outcome variables measured correctly using instruments/measurements that had been trialled, piloted or published previously?	1	0	1	1
10. Is it clear what was used to determined statistical significance and/or precision estimates? (e.g., p-values, confidence intervals)	1	1	1	1
11. Were the methods (including statistical methods) sufficiently described to enable them to be repeated?	1	1	1	1
12. Were the basic data adequately described?	1	1	1	1

13. Does the response rate raise concerns about non-response bias?	1	1	1	1
14. If appropriate, was information about non-responders described?	1	1	1	1
15. Were the results internally consistent?	1	1	1	1
16. Were the results presented for all the analyses described in the methods?	1	1	1	1
17. Were the authors' discussions and conclusions justified by the results?	1	1	1	1
18. Were the limitations of the study discussed?	1	1	1	1
19. Were there any funding sources or conflicts of interest that may affect the authors' interpretation of the results?	1	1	1	1
20. Was ethical approval or consent of participants attained?	1	1	1	1
**Total**	**18**	**18**	**19**	**19**

Note that 1 is given for low risk of bias and 0 is given for high risk of bias.

Cochrane Tool to Assess Risk of Bias in Cohort Studies

**Article ID:**	**2**	**3**
1. Was selection of exposed and non-exposed cohorts drawn from the same population?	4	4
2. Can we be confident in the assessment of exposure?	3	3
3. Can we be confident that the outcome of interest was not present at start of study	3	3
4. Did the study match exposed and unexposed for all variables that are associated with the outcome of interest or did the statistical analysis adjust for these prognostic variables?	4	4
5. Can we be confident in the assessment of the presence or absence of prognostic factors?	3	3
6. Can we be confident in the assessment of outcome?	3	3
7. Was the follow up of cohorts adequate?	3	4
8. Were co-Interventions similar between groups?	4	4
**Total**	**27**	**28**

Note that 4 is given for "Definitely Yes" (low risk of bias), 3 is given for "probably yes", 2 is given for "probably no", and 0 is given for "No" (high risk of bias).
